# Characterization and genome annotation of a newly detected bacteriophage infecting multidrug-resistant *Acinetobacter baumannii*

**DOI:** 10.1007/s00705-019-04213-0

**Published:** 2019-03-21

**Authors:** Zichen Yang, Xinzhu Liu, Yunlong Shi, Supeng Yin, Wei Shen, Jing Chen, Yu Chen, Yajie Chen, Bo You, Yali Gong, Xiaoqiang Luo, Cheng Zhang, Zhiqiang Yuan, Yizhi Peng

**Affiliations:** 1 State Key Laboratory of Trauma, Burns and Combined Injury, Institute of Burn Research, Southwest Hospital, Chongqing, China; 2 Department of microbiology, College of Basic Medicin, Third Military Medical University (Army Medical University), Chongqing, China

## Abstract

A novel virulent bacteriophage, φAbp2, infecting multidrug-resistant (MDR) *Acinetobacter baumannii* was isolated from the wastewater of a sewage management centre at Southwest Hospital, China. Transmission electron microscopy and phylogenetic analysis revealed that φAbp2 belongs to the subfamily *Peduovirinae*. A one-step growth curve demonstrated that φAbp2 had a latent period of 15 min, a lysis period of 35 min, and a burst size of 222 particles per infected host cell. Moreover, φAbp2 showed a relatively broad host range in local *A. baumannii*, and it also exhibited tolerance over a wider range of thermal and pH conditions. Genomic sequencing revealed that φAbp2 has a circular double-stranded DNA genome with no sequence similarity to our previously isolated φAbp1. Eighty-eight putative open reading frames (ORFs) encoding 41 proteins of known function and 47 of unknown function were identified, and the G/C content was 37.84%. φAbp2 is a new member of the subfamily *Peduovirinae* of the family *Myoviridae.* Its genome sequence is very similar to that of the *A. baumannii* phage LZ35.

**Electronic supplementary material:**

The online version of this article (10.1007/s00705-019-04213-0) contains supplementary material, which is available to authorized users.

## Introduction

*Acinetobacter baumannii* is a growing threat that is responsible for numerous healthcare-associated infections, such as burn and wound infections [1]. It is among the leading causes of infections of the respiratory and urinary tracts, secondary meningitis, and sepsis [2]. Some strains of this widespread Gram-negative pathogen have been reported recently to be resistant to nearly all known antibiotics, and there is therefore an urgent need to find alternative treatments for these infections [3, 4].

In the middle of the 1910s, it was suggested that bacteriophages could be used successfully for treatment of human infections [5]. In recent decades, several reports have revealed the existence of multidrug-resistant (MDR) *A. baumannii* strains with a high degree of resistance to β-lactam antibiotics, including cephalosporins and penicillins [6]. To combat MDR *A. baumannii* isolates, phages are now being considered as alternatives to antibiotics, a century after their discovery [7]. In recent decades, there has been increasing evidence of the feasibility of using phage therapy to treat drug-resistant bacterial infections [8, 9]. Indeed, not only can active phage be applied directly in the clinic [10], but new potential phage-derived antimicrobial agents are being identified and certified [11]. Thus, bacteriophage therapy is a potential strategy to fight MDR *A. baumannii*. However, problems including narrow host range, unclear host-phage interactions, and safety issues largely limit its clinical application.

Recently, we performed a national study of MDR *A. baumannii* epidemiology in which a number of bacteriophages were screened and preserved. A novel phage with strong lytic efficiency in MDR *A. baumannii* was isolated from wastewater from the intensive care unit of a burn treatment centre in southwestern China. Sequence analysis showed that this phage was completely different from our previous reported *A. baumannii* φAbp1 [12], and it was therefore named φAbp2. In this article, we describe the characterization and genome annotation of the phage φAbp2, which will provide important information for its further study and application.

## Materials and methods

### Bacterial preparations

All of the MDR *A. baumannii* strains were collected previously for an epidemiology study and stored at the Institute of Burn Research, Southwest Hospital, China. MDR *A. baumannii* cultures were inoculated at a dilution of 1:100 and grown aerobically overnight at 37 °C in LB (Luria-Bertani) broth or on LB solid medium.

### Antibiotic susceptibility tests (ASTs)

Antibiotic susceptibility tests of clinically important bacteria and fungi were performed and interpreted according to the criteria of the Clinical and Laboratory Standards Institute [13] for the corresponding year, and the manufacturers’ instructions were followed for the use of antibiotics.

To assess the resistance of pathogens to other antibiotics, the K-B disk diffusion method was applied. Nine antibiotics were selected: piperacillin, cefoperazone/sulbactam, sulfamethoxazole, ceftazidime, cefotaxime, imipenem, gentamicin, ciprofloxacin, and tigecycline (Oxoid, UK). Polymyxin B was not included in antibiotic susceptibility tests because a preliminary test showed no resistance among the collected strains (Table S1).

### Phage isolation and preparation

A conventional screening method was used for bacteriophage isolation [14]. A sewage water sample (500–1000 ml) that had not been disinfected was collected from the sewage management centre of Southwest Hospital. MDR *A. baumannii* was then cultured from roughly sterilized sewage, and the culture was filtered through a 0.22-μm membrane to collect bacteriophages, after which the sample was centrifuged at 13000 × *g* for 15 min at room temperature. The double-layer agar method was applied to identify target bacteriophages [14]. Briefly, 10 μl of supernatant and 100 μl of a MDR *A. baumannii* strain were combined for a 10-minute absorption period and then mixed with 3 ml of melted 0.7% soft agar. The mixture was then plated on an LB agar plate and incubated at 37 °C overnight. Clear plaques were picked aseptically and suspended in 500 μl of liquid LB medium. The process was repeated three times to isolate the phage. A new single-clone lytic bacteriophage plaque was picked and resuspended at 180 rpm in 6 ml of TM solution (10 mM Tris-HCl, 1 mM EDTA, [pH 8.0]) from the potentially inhomogeneous bacteriophage mixture. The SM supernatant containing the target bacteriophage was filtered through a 0.22-μm membrane and stored at 4 °C. The lysed strains were excluded from the next round of bacteriophage isolation to avoid spectrum overlap with previously isolated bacteriophages, as described previously [15].

To obtain φAbp2 particles on a large scale, single phage plaques were suspended and incubated overnight at 37 °C with shaking at 160 rpm. Then, DNase I and RNase A were added to the culture to a final concentration of 1 μg/ml, and incubation was continued at 37 °C for 30 min. NaCl was added at a concentration of 5.84 g/100 ml, mixed and dissolved, and the sample was then immersed in an ice bath for 1 h. The sample was centrifuged at 10000 × *g* for 10 min, and the supernatant was collected. Solid PEG 8000 (Oxoid, UK) was added to a final concentration of 10% (w/v), mixed and dissolved, and the sample was incubated in an ice bath overnight to let the phage pellet form a precipitate, which was collected by centrifugation at 12000 × *g* for 10 min at 4 °C, and the supernatant was discarded. Next, 2 ml per 100 ml of the original bacterial solution was added, adding TM solution to suspend the precipitate. Then, an equal volume of chloroform was added to the TM solution with shaking for 30 s, the sample was centrifuged 5000 × *g* for 10 min, and the upper aqueous phase was collected. The extract was again extracted once with an equal volume of chloroform to obtain crude granules of φAbp2.

### Transmission electron microscopy (TEM)

The purified phage suspension was dropped onto a copper grid surface and negatively stained with 2% uranyl acetate for approximately 15 s, and the excess stain was removed immediately. To examine the morphology of φAbp2, the copper grid was inspected under a 120-kV transmission electron microscope (JEM-1400 Plus, JEOL LTD, Japan).

### Thermal and pH stability

Following our previously described method [9], the stability (titre) of φAbp2 under different thermal and pH conditions was tested. Briefly, the purified phage was incubated at different temperatures (-80, -20, 4, 20, 40, 60 and 80 °C) for 48 h and at various pH levels (from 1 to 14) for 1 h. Then, the double-layer agar method was used to determine the titre of the incubated bacteriophages every 1 h. Both thermal and pH stability tests were performed in triplicate.

### One-step growth curve

For one-step growth experiments, MDR *A. baumannii* were infected with φAbp2 at a multiplicity of infection (MOI) of 0.1 using a 10-min adsorption time. Samples were centrifuged at 13,000 × *g* for 30 s to clear unabsorbed phages from the supernatants. The procedure was performed twice, using LB medium for washing. Then, the *A. baumannii* pellets with adsorbed φAbp2 were resuspended in 4 ml of LB liquid medium. After that, the cultures were grown at 37 °C with shaking at 160 rpm, during which samples were collected at 5- or 10-min intervals up to 100 min. The number of φAbp2 particles was immediately determined by the previously described double-layer agar method. This measurement was performed in triplicate.

### Host range determination and efficiency of plating

The host range of φAbp2 was determined using a spotting method as described previously [16]. In brief, 60 strains of MDR *A. baumannii* from different locations were included for host range testing (Table S2). All MDR *A. baumannii* strains were incubated overnight at 37 °C with φAbp2 to allow cell lysis. Plaques were inspected for lytic activity based on their clarity and transparency. The host range determination was performed in triplicate.

A more thorough assessment of productive infection as defined by the efficiency of plating (EOP) was conducted. Each strain was tested three times with each of four different dilutions of φAbp2 that had resulted in lysis in the host range determination. This was performed under the same conditions as in the spot assays. The phage lysates were diluted 10^6^- to 10^9^-fold. The plates were incubated overnight at 30 °C, and the number of plaque-forming units (PFU) was determined for each combination. When the 10^6^ dilution did not result in any plaques, a lower dilution was tried afterwards to verify that the EOP was lower than 0.001. Finally, the EOP was calculated (average PFU on target bacteria/average PFU on host bacteria) along with the standard deviation for the three measurements.

### DNA extraction, sequencing and genome analysis

Phage genomic DNA was extracted using the phenol-chloroform protocol [17]. Briefly, crude granules of φAbp2 were lysed by the addition of proteinase K (100 mg/ml) at 37 °C in water for 30 min. Then, EDTA (0.5 mM, pH 8.0) was added to stop the digestion of the φAbp2 sample. An equal volume of phenol was added to the mixture, which was then centrifuged at 5,000 × *g* (4 °C) for 10 min to remove debris. This step was repeated for a second time with chloroform. Then, the supernatant was incubated overnight with isoamyl alcohol at -20 °C to precipitate the DNA. After washing three times with cold 75% ethanol, the φAbp2’ genomic DNA was dissolved in TE buffer, quantified using a NanoDrop spectrophotometer (Thermo Scientific, Waltham, MA, USA) and sequenced on an Illumina HiSeq 2500 sequencer (San Diego, CA, USA) with a 2 × 100-bp read length.

Through sequencing the cloned fragments of the φAbp2 genome, more than 7,300-fold coverage was obtained. Then, the short reads were assembled using SOAPdenovo (http://sourceforge.net/projects/soapdenovo2/fles/SOAPdenovo2/) to the genome sequence. The assembled whole genome sequence was searched against the current nucleotide databases (http://www.ncbi.nlm.nih.gov/) using the Basic Local Alignment Search Tool (BLAST). Putative protein-encoding open reading frames (ORFs) were jointly predicted using online bioinformatic tools, including GeneMark (http://topaz.gatech.edu/GeneMark/), FgenesB (http://linux1.softberry.com/berry.phtml?topic=fgenesb&group=programs&subgroup=gfindb), Rast (http://rast.nmpdr.org/) and Glimmer (http://ccb.jhu.edu/software/glimmer/index.shtml). Then, the intersecting predicted ORFs from different databases and algorithms were examined manually. Protein BLAST (BLASTP) (http://www.ncbi.nlm.nih.gov/BLAST/) was applied to match putative proteins sharing similarities with the predicted phage ORFs. Phylogenetic analysis of phage large terminase subunit sequences was performed using ClustalW (https://www.ebi.ac.uk/Tools/msa/clustalw2/) and MEGA 6 (https://www.megasoftware.net/). The annotated φAbp2 genome sequence was submitted to the NCBI database through Sequin (https://www.ncbi.nlm.nih.gov/Sequin/) under accession number MF346584.1.

## Results

### Morphology of the phage φAbp2

φAbp2 targeting an MDR *A. baumannii* strain was screened from the sewage management centre at Southwest Hospital. According to the TEM image of Abp2, the isometric polyhedral head was 85.5 ± 3.4 nm in diameter, whereas the contractile tail was 86.4 ± 3.4 nm in length and 23.3 ± 2.4 nm in width (Supplementary Fig S1). Thus, φAbp2 was designated to the family *Myoviridae*, order *Caudovirales*, following the current guidelines of the ICTV (International Committee on Taxonomy of Viruses, http://ictv.global/taxonomyReleases.asp).

### Life cycle of phage φAbp2

The life cycle of φAbp2 was investigated by performing a one-step growth experiment. The duration of the latent period was short (~ 15 min at 37 °C) and was followed by a lysis period that lasted 50 min. The burst size was 222 phage particles per infected cell after 100 min (Supplementary Fig S2). Moreover, φAbp2 was stable at temperatures ranging from -80 to 20 °C. However, the viral titres decreased slightly at 40 °C and were reduced dramatically at 60 and 80 °C (Supplementary Fig S3A). In addition, the pH tolerance of φAbp2 was stable over a broad range (from 5.0–10.0). However, the titre dropped significantly at pH 4.0 and pH 11.0 (Supplementary Fig S3B).

### Host range

Sixty MDR *A. baumannii* strains from different locations were selected for a phage host-range test to determine the lytic range of phage φAbp2. All of the local MDR *A. baumannii* strains tested were vulnerable to phage φAbp2 (Table 1), while none of the allopatric strains were lysed except for one strain from XiAn and three strains from Nanjing (Supplementary Table S2), indicating that φAbp2 is capable of lysing local strains but is limited to a specific geographic area.

**Table 1:** Host range summary of phage AbP2

Bacterial source	Number lytic (n/10)	Percentage lytic	EOP^^^
BICU^*^, Southwest Hospital, Chongqign	10/10	100%	1.0
XiAn	2/10	20%	0.3
He Nan province	0/10	0%	N/A^#^
BICU, Honghui Hospital, Shenzhen	0/10	0%	N/A
Ji Lin province	0/10	0%	N/A
Nanjing	4/10	40%	0.5

*BICU, burn intensive care unit

^EOP, average efficiency of plating

#N/A not tested; phage AbP2 did not lyse the bacterium

### Genome annotation and analysis

**Fig. 1:**
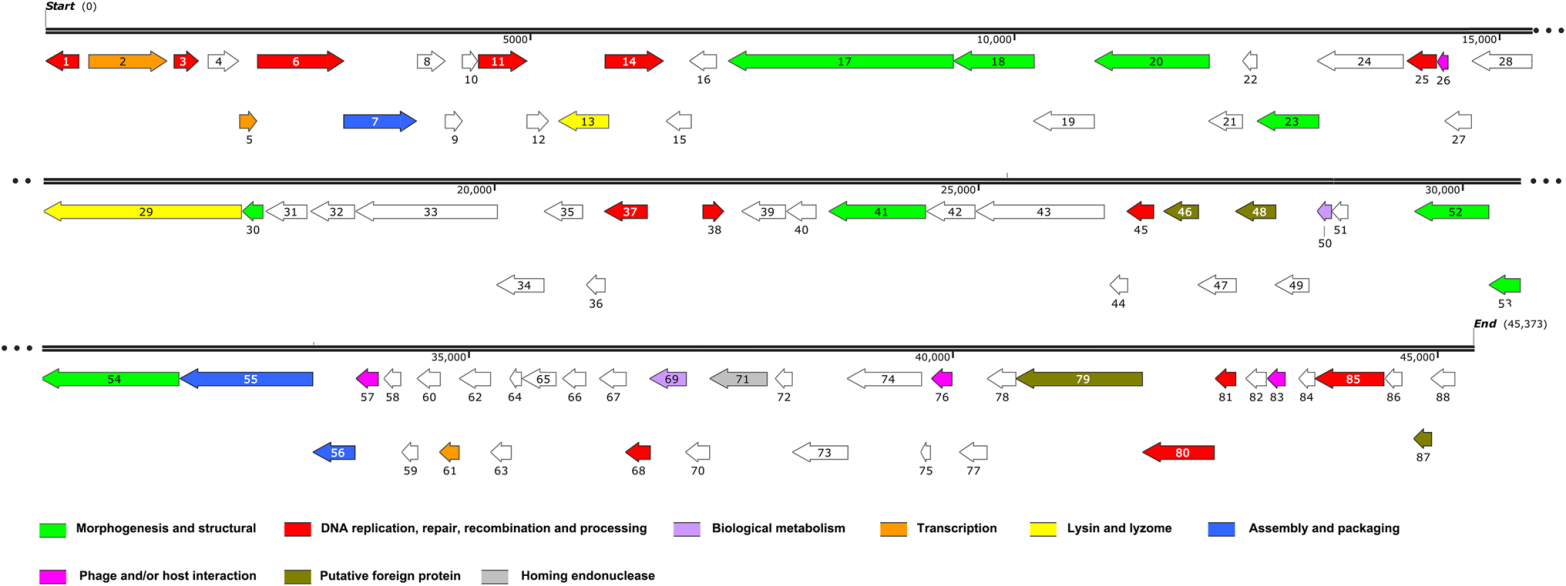
Detailed φAbp2 genome annotation. Predicted genes are represented by arrows with their direction of transcription indicated by the direction of each arrow. Different colours indicate different functional groups of genes

High-throughput sequencing reads were assembled into a completely closed, circular genome sequence using SOAPdenovo. The circularity of the phage genome was confirmed by restriction endonuclease mapping (data not shown). This double-stranded DNA genome of φAbp2 consisted of 45,373 bp, with a GC content of 37.84% (Fig. 1). BLASTn analysis of the whole genome sequence revealed that the genome of φAbp2 shared 0% sequence identity with our previously isolated φAbp1, suggesting that they are unrelated to each other. However, the φAbp2 genome sequence exhibited 93% nucleotide sequence identity, with 71% coverage to *A. baumannii* phage LZ35, with a number of ORFs nevertheless differing significantly (Table S3). The genome was found to contain 88 putative ORFs (Fig. 1). ORFs encoding proteins with known functions were classified into several groups: those associated with morphogenesis and structure, DNA replication, repair, recombination and processing, biological metabolism, transcription, lysis, assembly and packaging, phage and/or host interaction, putative foreign proteins, and homing endonucleases (Fig. 1).

**Fig. 2:**
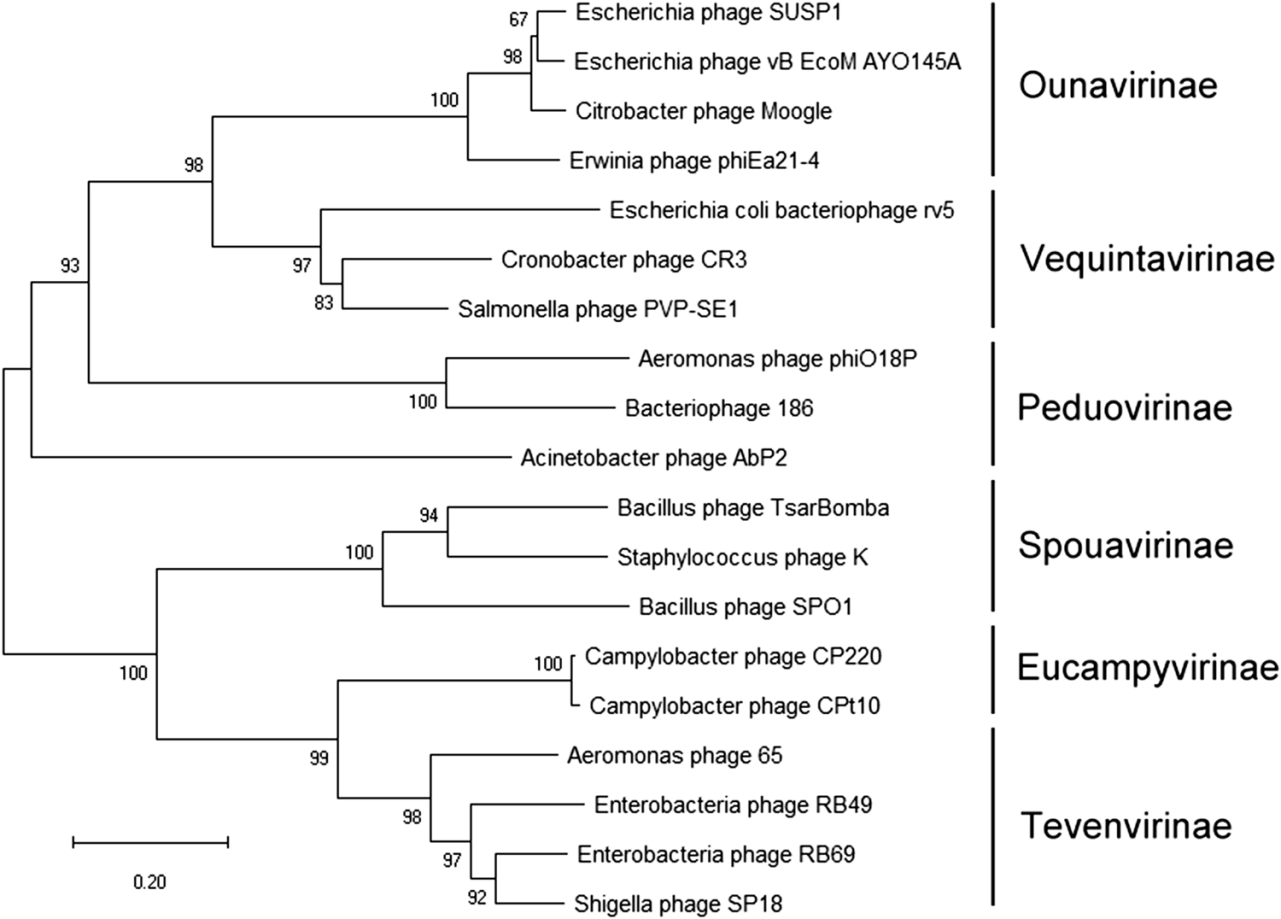
Phylogenetic analysis of the φAbp2 large terminase subunit (ORF55). The amino acid sequences of the large terminase subunits were compared using MEGA6, and a phylogenetic tree was generated by the neighbour-joining method with 1000 bootstrap replicates

Structure- and assembly-associated proteins identified in BLASTn and BLASTp analysis include the tail fibre protein (ORF17, 18 and possibly ORF30), baseplate-associated protein (ORF20 and 23), putative capsid protein (ORF41), head protein (ORF 52) and portal protein (ORF54). Genes encoding DNA replication, repair, recombination and processing modules, including transposase (ORF1 and 68), GTPase (ORF3), recombinase (ORF6), nucleoside triphosphate pyrophosphohydrolase (ORF11), nuclease (ORF14), methyltransferase (ORF38 and 45), helicase (ORF79), primosomal protein (ORF81), and DNA-binding protein (ORF85), were distributed throughout the whole φAbp2 phage genome (Fig. 2, Table S3). Among these sequences, 41 ORFs encoded proteins with high levels of similarity to phage proteins with similar functions, while 47 undefined ORFs were also identified. We identified ORFs with biological metabolism, transcription, lysin and lyzome, assembly and packaging, phage and/or host interaction, and homing endonuclease functions (Fig. 1), but four ORFs (ORF46, 48, 80, and 87) were predicted to encode putative foreign proteins, although the level of sequence identity was only 24–42% (Table S3).

### Phylogenetic analysis

To further classify φAbp2, we aligned the amino acid sequence of the terminase large subunit proteins (encoded by ORF55) with those from other phages of the family *Myoviridae,* using MEGA6 software. A neighbour-joining tree generated from the alignments revealed that φAbp2 clustered with *Peduovirinae* phages such as bacteriophage 186 and *Aeromonas* phage phiO18P (Fig. 2). The characteristics of the phylogenetic tree and its unique genome features indicate that φAbp2 is a new member of the family *Myoviridae*, order *Caudovirales*.

## Discussion

In this study, we present the characterization and genomic annotation of φAbp2, a lytic phage from a clinical MDR *A. baumannii* isolate with strong lytic ability and a wider host range than those of our previously screened bacteriophages [14].

TEM and phylogenetic analysis indicated that phage φAbp2 should be assigned to the subfamily *Peduovirinae* based on its morphological characteristics and its phylogenetic relationship to phages of the family *Myoviridae*. Based on its morphology and genome features, phage φAbp2 was determined to be a new member of the subfamily *Peduovirinae* of the family *Myoviridae*, order *Caudovirales*.

One-step growth curve analysis revealed a 15-min latent period, a 35-min lysis period and a burst size of 222 phage particles per infected host cell, which is smaller than that of φAbp1 [14]. Compared with our previously isolated φAbp1, φAbp2 had a smaller burst size [14] but a wider range of pH and thermal tolerance [9] as well as a broader host range among local MDR *A. baumannii* strains, making φAbp2 a more suitable candidate than φAbp2 for clinical application.

The high mutation rate of bacteriophages makes it difficult to identify gene functions [18], and this lack of information limits their application and causes uncertainty regarding their safety [19, 20]. There are many similarities between the genes of φAbp2 and those of previously identified bacteriophages. The genome sequence of phage φAbp2 showed the highest nucleotide sequence identity to that of *A. baumannii* phage LZ35. Functional prediction revealed 88 possible ORFs, of which only 41 showed relatively high amino acid sequence similarity to other hypothetical proteins. The other 47 (53.4%) ORFs could not be annotated, indicating that φAbp2 is a unique phage. Moreover, the lack of clarity about the function of many of the genes creates obstacles to broadening the narrow host spectrum of this bacteriophage. Previous studies have identified host-range determinants such as gp17 in T7 phage and gp37 and gp3 in T4-like phages [21, 22], but none of the currently known host range determinants showed similarity to the ORFs of φAbp2, suggesting that the unannotated ORFs might hold the key to the specific host range characteristics of φAbp2.

The putative foreign genes of φAbp2 (ORF46, 48, 80, and 87) showed a degree of similarity to genes of non-microbial organisms suggesting that this phage can carry foreign genes and possibly even human genes. It is proposed that φAbp2 may interact directly or indirectly with these organisms, since phages might acquire foreign genes to allow them to produce soluble factors [23]. To determine whether phage φAbp2 might be temperate, we searched for lysogenic phage genes, but found only one possible lysogenic gene (ORF30). Although no known lysogenic factors, including excisionase or anti-repressor proteins [24], were found during the genome annotation of φAbp2, the predicted proteins with unknown functions might nevertheless have lysogen-related functions. The presence of ORFs (ORF46 and 80) with similarity to two genes of *Homo sapiens* suggests that these genes might have been acquired from humans. The application of φAbp2 to humans might lead to complications, including immune effects, as phages have been shown recently to interact directly with somatic cells [25–27]. The presence of foreign gene fragments reminds us that the clinical use of phages will require caution, and it might be more suitable to use “clean, simple” engineered phages in clinical practice.

## Conclusion

We isolated and sequenced the genome of phage φAbp2, which has a broad host lysis spectrum in native strains and broad tolerance, which improves its prospects for clinical application. Its host spectrum requires further research at the genetic level. A putative endolysin gene (ORF13) was identified in the phage φAbp2 genome. Future work should examine potential applications of φAbp2 in the treatment of MDR *A. baumannii* and the interaction between φAbp2 and its hosts.

## Electronic supplementary material

Below is the link to the electronic supplementary material.

Morphology of φAbp2 by transmission electron microscopy. The isometric polyhedral head of φAbp2 is approximately 85.5 ± 3.4 nm in diameter. The contractile tail is approximately 86.4 ± 3.4 nm in length and 23.3 ± 2.4 nm in width. Nine repeated measurements of seven phage bodies were calculated, and the data are expressed as the mean ± SDs. The scale bar represents 200 nm. Magnification: × 150,000 (TIFF 320 kb)

Thermal and pH stability of φAbp2. The data are expressed as the mean ± SDs (A, temperature; B, pH) (TIFF 1529 kb)

One-step growth curve of φAbp2 in an MDR *A****. baumannii*host.** φAbp2 had a short latent period (~ 15 min) with an average burst size of 222 phage particles per infected cell after 100 min. The data are expressed as the mean ± SDs (TIFF 60 kb)

Summary of the ASTs (XLSX 14 kb)

Host range details for φAbp2 (DOCX 22 kb)

Genome annotation data (DOCX 28 kb)
