# Supplementary figures and images for: Characterization and genome annotation of a newly detected bacteriophage infecting multidrug-resistant Acinetobacter baumannii
Source: Arch Virol. 2019 Mar 21;164(6):1527–33. doi: 10.1007/s00705-019-04213-0 (PMC6526140; doi:10.1007/s00705-019-04213-0)

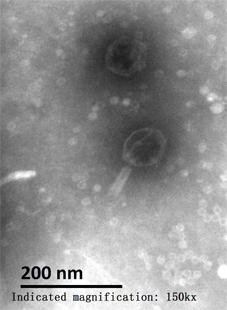

Supplement: Supplementary file 1 — Morphology of φAbp2 by transmission electron microscopy. The isometric polyhedral head of φAbp2 is approximately 85.5 ± 3.4 nm in diameter. The contractile tail is approximately 86.4 ± 3.4 nm in length and 23.3 ± 2.4 nm in width. Nine repeated measurements of seven phage bodies were calculated, and the data are expressed as the mean ± SDs. The scale bar represents 200 nm. Magnification: × 150,000 (TIFF 320 kb) [file 705_2019_4213_MOESM1_ESM.tif]

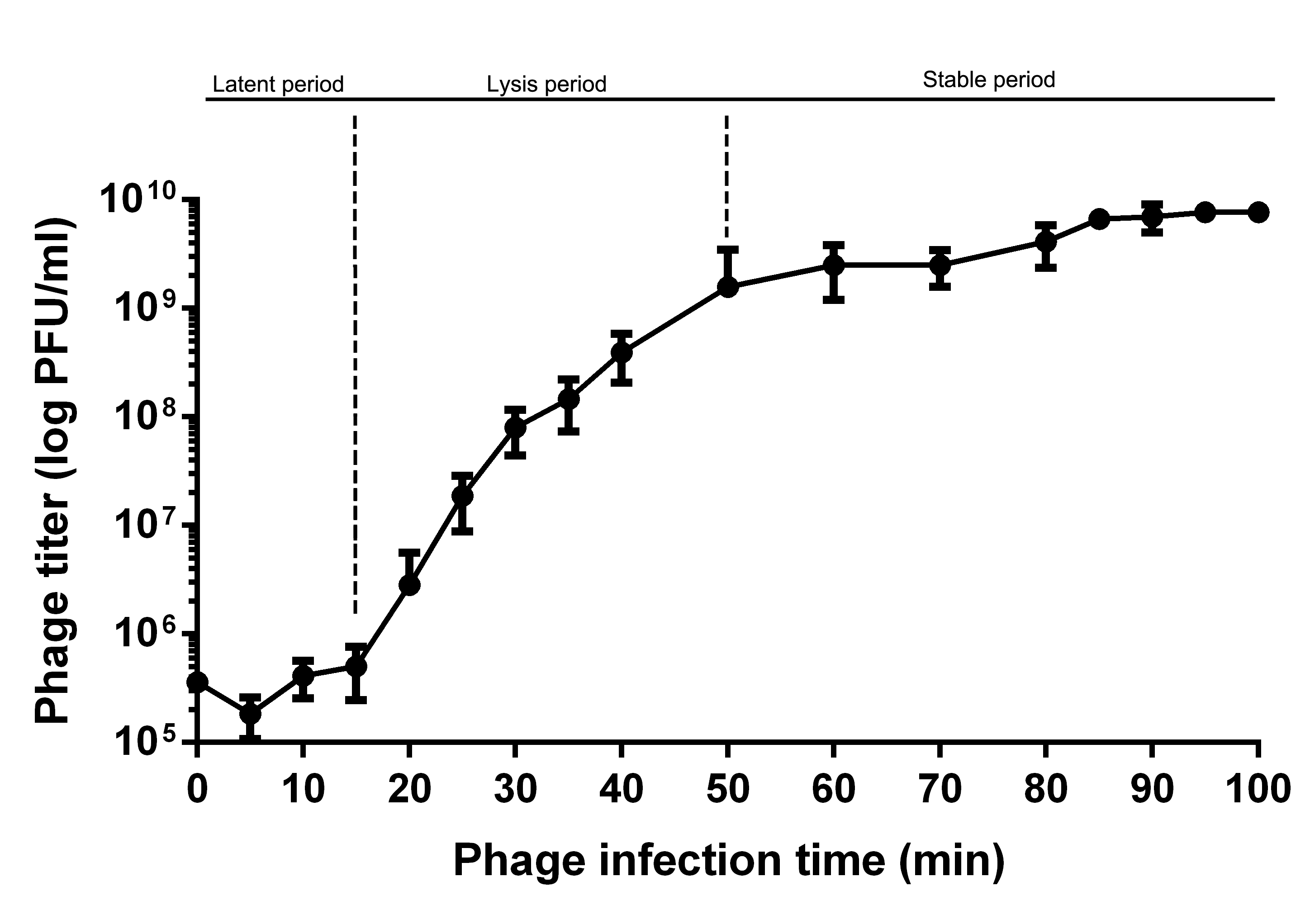

Supplement: Supplementary file 2 — Thermal and pH stability of φAbp2. The data are expressed as the mean ± SDs (A, temperature; B, pH) (TIFF 1529 kb) [file 705_2019_4213_MOESM2_ESM.tif]
