# Supplementary material for: Characterization and genome annotation of a newly detected bacteriophage infecting multidrug-resistant Acinetobacter baumannii
Source: Arch Virol. 2019 Mar 21;164(6):1527–33. doi: 10.1007/s00705-019-04213-0 (PMC6526140; doi:10.1007/s00705-019-04213-0)
Supplement: Supplementary file 5 — Host range details for φAbp2 (DOCX 22 kb) [file 705_2019_4213_MOESM5_ESM.docx]

**Supplementary Table S2. Host range of phage AbP2.**

| Strain | Lytic activity | EOP^^^ | Source | Strain | Lytic activity | EOP | Source |
| --- | --- | --- | --- | --- | --- | --- | --- |
| 201407074 | +++ | 1.0 | BICU*, Southwest Hospital, Chongqign | G001 | - | N/A | BICU, Honghui Hospital, Shenzhen |
| 201407072 | +++ | 1.0 | BICU, Southwest Hospital, Chongqign | G002 | - | N/A | BICU, Honghui Hospital, Shenzhen |
| 201407071 | +++ | 1.0 | BICU, Southwest Hospital, Chongqign | G003 | - | N/A | BICU, Honghui Hospital, Shenzhen |
| 201407070 | +++ | 1.0 | BICU, Southwest Hospital, Chongqign | G004 | - | N/A | BICU, Honghui Hospital, Shenzhen |
| 201407069 | +++ | 1.0 | BICU, Southwest Hospital, Chongqign | G005 | - | N/A | BICU, Honghui Hospital, Shenzhen |
| 201402007 | +++ | 1.0 | BICU, Southwest Hospital, Chongqign | G006 | - | N/A | BICU, Honghui Hospital, Shenzhen |
| 201402823 | +++ | 1.0 | BICU, Southwest Hospital, Chongqign | G007 | - | N/A | BICU, Honghui Hospital, Shenzhen |
| 201402006 | +++ | 1.0 | BICU, Southwest Hospital, Chongqign | G008 | - | N/A | BICU, Honghui Hospital, Shenzhen |
| 201407074 | +++ | 1.0 | BICU, Southwest Hospital, Chongqign | G009 | - | N/A | BICU, Honghui Hospital, Shenzhen |
| 201407074 | +++ | 1.0 | BICU, Southwest Hospital, Chongqign | G010 | - | N/A | BICU, Honghui Hospital, Shenzhen |
| X001 | + | 0.3 | XiAn | J001 | - | N/A | Ji Lin province |
| X002 | - |  | XiAn | J002 | - | N/A | Ji Lin province |
| X003 | - |  | XiAn | J003 | - | N/A | Ji Lin province |
| X004 | - |  | XiAn | J004 | - | N/A | Ji Lin province |
| X005 | - |  | XiAn | J005 | - | N/A | Ji Lin province |
| X006 | - |  | XiAn | J006 | - | N/A | Ji Lin province |
| X007 | - |  | XiAn | J007 | - | N/A | Ji Lin province |
| X008 | - |  | XiAn | J008 | - | N/A | Ji Lin province |
| X009 | + | 0.3 | XiAn | J009 | - | N/A | Ji Lin province |
| X010 | - | N/A^#^ | XiAn | J010 | - | N/A | Ji Lin province |
| H001 | - | N/A | He Nan province | N001 | - | N/A | Nanjing |
| H002 | - | N/A | He Nan province | N002 | - | N/A | Nanjing |
| H003 | - | N/A | He Nan province | N003 | - | N/A | Nanjing |
| H004 | - | N/A | He Nan province | N004 | ++ | 0.5 | Nanjing |
| H005 | - | N/A | He Nan province | N005 | ++ | 0.5 | Nanjing |
| H006 | - | N/A | He Nan province | N006 | ++ | 0.5 | Nanjing |
| H007 | - | N/A | He Nan province | N007 | ++ | 0.5 | Nanjing |
| H008 | - | N/A | He Nan province | N008 | - | N/A | Nanjing |
| H009 | - | N/A | He Nan province | N009 | - | N/A | Nanjing |
| H010 | - | N/A | He Nan province | N010 | - | N/A | Nanjing |

*BICU, burn intensive care unit.

^^^EOP, Efficiency of plating.

^#^N/A, non-conducted.
