# Supplementary material for: Characterization and genome annotation of a newly detected bacteriophage infecting multidrug-resistant Acinetobacter baumannii
Source: Arch Virol. 2019 Mar 21;164(6):1527–33. doi: 10.1007/s00705-019-04213-0 (PMC6526140; doi:10.1007/s00705-019-04213-0)
Supplement: Supplementary file 6 — Genome annotation data (DOCX 28 kb) [file 705_2019_4213_MOESM6_ESM.docx]

**Table S1. Phage AbP2 gene annotation.** ^aA^mino acids (aa).

| ORFs | Start | Stop | Length (aa) | Function | aa identity % | Accession number | Best *e*-value |
| --- | --- | --- | --- | --- | --- | --- | --- |
| ABP2_001 | 344 | 3 | 342 | Putative transposase [Maledivibacter halophilus] | 33% | WP_079490418.1 | 0.016 |
| ABP2_002 | 446 | 1258 | 813 | Putative transcriptional regulator [Acinetobacter phage WCHABP12] | 96% | ARB06770.1 | 2e-108 |
| ABP2_003 | 1325 | 1582 | 258 | Dynamin family GTPase Msp1 [Schizosaccharomyces pombe] | 36% | NP_596452.1 | 0.45 |
| ABP2_004 | 1675 | 2007 | 333 | Hypothetical protein |  |  |  |
| ABP2_005 | 2007 | 2189 | 183 | HTH-type transcriptional regulator MlrA [Enterobacter cloacae] | 72% | WP_102749964.1 | 0.032 |
| ABP2_006 | 2186 | 3085 | 900 | Recombinase [Thermovibrio guaymasensis] | 99% | WP_121172002.1 | 0.0 |
| ABP2_007 | 3082 | 3837 | 756 | Translocation protein TolB precursor [Thermovibrio guaymasensis] | 98% | WP_121172004.1 | 0.0 |
| ABP2_008 | 3838 | 4131 | 294 | Hypothetical protein |  |  |  |
| ABP2_009 | 4128 | 4310 | 183 | Hypothetical protein |  |  |  |
| ABP2_010 | 4307 | 4468 | 162 | Hypothetical protein |  |  |  |
| ABP2_011 | 4471 | 4977 | 507 | Putative nucleoside triphosphate pyrophosphohydrolase [Acinetobacter phage WCHABP1] | 100% | ARQ94810.1 | 3e-126 |
| ABP2_012 | 4970 | 5200 | 231 | Hypothetical protein |  |  |  |
| ABP2_013 | 5811 | 5299 | 513 | Putative endolysin [Acinetobacter phage WCHABP1] | 100% | AST13128.1 | 8e-124 |
| ABP2_014 | 5773 | 6384 | 612 | Putative nucleases NUMOD4 motif protein | 100% | AYP69081.1 | 5e-135 |
| ABP2_015 | 6667 | 6404 | 264 | Hypothetical protein |  |  |  |
| ABP2_016 | 6929 | 6651 | 279 | Hypothetical protein |  |  |  |
| ABP2_017 | 9377 | 7047 | 2331 | Tail fiber protein [Acinetobacter phage WCHABP1] | 78% | ARQ94726.1 | 2e-77 |
| ABP2_018 | 10,209 | 9379 | 831 | Putative tail fiber protein [Acinetobacter phage YMC11/12/R2315] | 95% | YP_009203603.1 | 0.0 |
| ABP2_019 | 10,828 | 10,202 | 627 | Hypothetical protein |  |  |  |
| ABP2_020 | 12,012 | 10,828 | 1185 | Baseplate J-like protein [Thermovibrio guaymasensis] | 99% | RKQ59850.1 | 0.0 |
| ABP2_021 | 12,362 | 12,009 | 354 | Hypothetical protein |  |  |  |
| ABP2_022 | 12,505 | 12,359 | 147 | Hypothetical protein |  |  |  |
| ABP2_023 | 13,152 | 12,508 | 645 | Putative baseplate assembly protein [Acinetobacter phage WCHABP1] | 98% | ARQ94731.1 | 4e-159 |
| ABP2_024 | 14,023 | 13,133 | 891 | Hypothetical protein |  |  |  |
| ABP2_025 | 14,366 | 14,058 | 309 | 2'-5' RNA ligase [Acidomonas methanolica NBRC 104435] | 43% | GAJ28859.1 | 0.002 |
| ABP2_026 | 14,454 | 14,347 | 108 | RHS repeat-associated core domain-containing protein [Desulfatibacillum alkenivorans DSM 16219] | 67% | SHL09759.1 | 0.039 |
| ABP2_027 | 14,726 | 14,451 | 276 | Hypothetical protein |  |  |  |
| ABP2_028 | 15,340 | 14,723 | 618 | Hypothetical protein |  |  |  |
| ABP2_029 | 17,396 | 15,348 | 2049 | Lysozyme like domain protein [Acinetobacter phage YMC-13-01-C62] | 96% | YP_009055475.1 | 0.0 |
| ABP2_030 | 17,611 | 17,399 | 213 | Putative tail-fiber protein / lysozyme protein [Acinetobacter phage LZ35] | 91% | YP_009055476.1 | 6e-48 |
| ABP2_031 | 18,066 | 17,641 | 426 | Hypothetical protein |  |  |  |
| ABP2_032 | 18,560 | 18,111 | 450 | Hypothetical protein |  |  |  |
| ABP2_033 | 20,036 | 18,573 | 1464 | Hypothetical protein |  |  |  |
| ABP2_034 | 20,520 | 20,026 | 495 | Hypothetical protein |  |  |  |
| ABP2_035 | 20,915 | 20,517 | 399 | Hypothetical protein |  |  |  |
| ABP2_036 | 21,146 | 20,958 | 189 | Hypothetical protein |  |  |  |
| ABP2_037 | 21,580 | 21,143 | 438 | Putative RNA polymerase [Acinetobacter phage WCHABP12] | 99% | ARB06827.1 | 3e-109 |
| ABP2_038 | 22,156 | 22,380 | 225 | Class I SAM-dependent methyltransferase [Halobacteriales archaeon QH_8_68_33] | 41% | PSP85218.1 | 2e-04 |
| ABP2_039 | 23,010 | 22,558 | 453 | Hypothetical protein |  |  |  |
| ABP2_040 | 23,328 | 23,020 | 309 | Hypothetical protein |  |  |  |
| ABP2_041 | 24,460 | 23,453 | 1008 | Putative capsid protein [Acinetobacter phage WCHABP12] | 99% | ARB06823.1 | 0.0 |
| ABP2_042 | 24,965 | 24,471 | 495 | Hypothetical protein |  |  |  |
| ABP2_043 | 26,303 | 24,975 | 1329 | Conserved protein of unknown function [Acinetobacter phage AP22] | 95% | YP_006383778.1 | 0.0 |
| ABP2_044 | 26,548 | 26,363 | 186 | Hypothetical protein |  |  |  |
| ABP2_045 | 26,813 | 26,538 | 276 | DNA (cytosine-5)-methyltransferase 1 [Roseibaca calidilacus] | 29% | CUX80688.1 | 0.031 |
| ABP2_046 | 27,274 | 26,912 | 363 | Trafficking protein particle complex subunit 10 isoform X7 [Homo sapiens] | 30% | XP_011528020.1 | 0.31 |
| ABP2_047 | 27,663 | 27,271 | 393 | Hypothetical protein |  |  |  |
| ABP2_048 | 28,078 | 27,656 | 423 | WD repeat-containing protein 20 [Danio rerio] | 24% | XP_003200833.1 | 0.19 |
| ABP2_049 | 28,421 | 28,068 | 354 | Hypothetical protein |  |  |  |
| ABP2_050 | 28,649 | 28,503 | 147 | Enamine deaminase RidA [Arcobacter marinus] | 39% | WP_099310619.1 | 0.003 |
| ABP2_051 | 28,814 | 28,650 | 165 | Hypothetical protein |  |  |  |
| ABP2_052 | 30,274 | 29,504 | 771 | Putative head protein [Acinetobacter phage YMC11/12/R2315] | 99% | YP_009203553.1 | 0.0 |
| ABP2_053 | 30,600 | 30,277 | 324 | Putative head protein |  |  |  |
| ABP2_054 | 32,020 | 30,593 | 1428 | Putative portal protein [Acinetobacter phage AP22] | 96% | YP_006383767.1 | 0.0 |
| ABP2_055 | 33,396 | 32,023 | 1374 | Terminase large subunit [Acinetobacter phage vB_KpnM_IME512] | 99% | AYP69032.1 | 0.0 |
| ABP2_056 | 33,836 | 33,396 | 441 | Terminase [Rodentibacter trehalosifermentans] | 60% | WP_077478716.1 | 7e-63 |
| ABP2_057 | 34,076 | 33,840 | 237 | RHS repeat-associated core domain protein [Acinetobacter baumannii 299505] | 29% | EXB78265.1 | 2.3 |
| ABP2_058 | 34,302 | 34,132 | 171 | Hypothetical protein |  |  |  |
| ABP2_059 | 34,480 | 34,313 | 168 | Hypothetical protein |  |  |  |
| ABP2_060 | 34,712 | 34,470 | 243 | Hypothetical protein |  |  |  |
| ABP2_061 | 34,906 | 34,709 | 198 | Global transcriptional activator Fis [Shewanella oneidensis MR-1] | 46% | NP_716031.1 | 6e-04 |
| ABP2_062 | 35,235 | 34,909 | 327 | Hypothetical protein |  |  |  |
| ABP2_063 | 35,450 | 35,235 | 216 | Hypothetical protein |  |  |  |
| ABP2_064 | 35,565 | 35,443 | 123 | Hypothetical protein |  |  |  |
| ABP2_065 | 35,909 | 35,562 | 348 | Hypothetical protein |  |  |  |
| ABP2_066 | 36,212 | 35,973 | 240 | Hypothetical protein |  |  |  |
| ABP2_067 | 36,634 | 36,353 | 282 | Hypothetical protein |  |  |  |
| ABP2_068 | 36,881 | 36,621 | 261 | ISPsy24, transposase orfB [Pseudomonas amygdali pv. mori str. 301020] | 55% | EGH26556.1 | 0.008 |
| ABP2_069 | 37,258 | 36,878 | 381 | Carbamoyl phosphate synthase large subunit [Deltaproteobacteria bacterium TMED58] | 41% | OUU32998.1 | 0.003 |
| ABP2_070 | 37,496 | 37,251 | 246 | Hypothetical protein |  |  |  |
| ABP2_071 | 38,085 | 37,501 | 585 | Putative HNH homing endonuclease |  |  |  |
| ABP2_072 | 38,350 | 38,177 | 174 | Hypothetical protein |  |  |  |
| ABP2_073 | 38,922 | 38,347 | 576 | Hypothetical protein |  |  |  |
| ABP2_074 | 39,680 | 38,919 | 762 | Hypothetical protein |  |  |  |
| ABP2_075 | 39,778 | 39,680 | 99 | Hypothetical protein |  |  |  |
| ABP2_076 | 40,002 | 39,790 | 213 | Putative bacteriophage-associated immunity protein [Acinetobacter phage IME-AB2] | 94% | AFV51531.1 | 1e-46 |
| ABP2_077 | 40,364 | 40,074 | 291 | Hypothetical protein |  |  |  |
| ABP2_078 | 40,660 | 40,361 | 300 | Hypothetical protein |  |  |  |
| ABP2_079 | 41,962 | 40,661 | 1302 | Replicative DNA helicase [Pseudomonas aeruginosa PAO1] | 31% | NP_253618.1 | 6e-55 |
| ABP2_080 | 42,705 | 41,962 | 744 | CREB-binding protein isoform b [Homo sapiens] | 34% | NP_001073315.1 | 0.003 |
| ABP2_081 | 42,928 | 42,716 | 213 | Primosomal protein DnaT [Escherichia coli str. K-12 substr. MG1655] | 38% | NP_418782.1 | 1e-05 |
| ABP2_082 | 43,244 | 43,032 | 213 | Hypothetical protein |  |  |  |
| ABP2_083 | 43,438 | 43,256 | 183 | Toxin-antitoxin system HicB family antitoxin [Xenorhabdus sp. KJ12.1] | 42% | WP_099110404.1 | 3e-05 |
| ABP2_084 | 43,742 | 43,575 | 168 | Hypothetical protein |  |  |  |
| ABP2_085 | 44,459 | 43,743 | 717 | Putative DNA-binding protein [Acinetobacter phage AP22] | 89% | YP_006383824.1 | 7e-163 |
| ABP2_086 | 44,642 | 44,469 | 174 | Hypothetical protein |  |  |  |
| ABP2_087 | 45,187 | 44,939 | 186 | Zinc finger protein 574 [Danio rerio] | 42% | NP_001164307.1 | 0.032 |
| ABP2_088 | 45,187 | 44,939 | 249 | Hypothetical protein |  |  |  |
